# Effect of Brine Concentrations on the Bacteriological and Chemical Quality and Histamine Content of Brined and Dried Milkfish

**DOI:** 10.3390/foods9111597

**Published:** 2020-11-03

**Authors:** Chiu-Chu Hwang, Yi-Chen Lee, Chung-Yung Huang, Hsien-Feng Kung, Hung-Hui Cheng, Yung-Hsiang Tsai

**Affiliations:** 1 Department of Hospitality Management, Yu Da University of Science and Technology, Miaoli 361027, Taiwan; 2 Department of Seafood Science, National Kaohsiung University of Science and Technology, Kaohsiung 811213, Taiwan; lionlee@nkust.edu.tw (Y.-C.L.); cyhuang@nkust.edu.tw (C.-Y.H.); 3 Department of Pharmacy, Tajen University, Pingtung 907391, Taiwan; khfeng@mail.tajen.edu.tw; 4 Mariculture Research Center, Fisheries Research Institute, Council of Agriculture, Tainan 724028, Taiwan; cheng.hunghui@msa.hinet.net

**Keywords:** histamine, dried milkfish, hygienic quality, brine-salting

## Abstract

In this research, the occurrence of hygienic quality and histamine in commercial brined and dried milkfish products, and the effects of brine concentrations on the quality of brined and dried milkfish, were studied. Brined and dried milkfish products (*n* = 20) collected from four retail stores in Taiwan were tested to investigate their histamine-related quality. Among them, five tested samples (25%, 5/20) had histamine contents of more than 5 mg/100 g, the United States Food and Drug Administration guidelines for scombroid fish, while two (10%, 2/20) contained 69 and 301 mg/100 g of histamine, exceeding the 50 mg/100 g potential hazard level. In addition, the effects of brine concentrations (0%, 3%, 6%, 9%, and 15%) on the chemical and bacteriological quality of brined and dried milkfish during sun-drying were evaluated. The results showed that the aerobic plate count (APC), coliform, water activity, total volatile basic nitrogen (TVBN), and histamine content values of the brined and dried milkfish samples decreased with increased brine concentrations, whereas those of salt content and thiobarbituric acid (TBA) increased with increasing brine concentrations. The milkfish samples prepared with 6% NaCl brine had better quality with respect to lower APC, TVBN, TBA, and histamine levels.

## 1. Introduction

Histamine is a biogenic amine in charge of histamine fish poisoning (HFP) or scombroid poisoning. Histamine fish poisoning is a food outbreak with allergy-like symptoms arising from ingesting mishandled scombroid fish that have high levels of histamine in their flesh [1]. Histamine is formed mainly through the decarboxylation of free histidine in fish muscles by histidine decarboxylases produced by a number of histamine-forming bacteria present in seafood [2]. HFP has occasionally been associated with the consumption of milkfish, marlin, mackerel, and tuna in Taiwan [2,3,4,5,6]. However, there is compelling evidence to implicate that other factors, such as other biogenic amines, can potentiate histamine toxicity, as spoiled fish containing histamine tends to be more toxic than the equivalent amount of pure histamine that is ingested orally [1,2]. Putrescine and cadaverine were shown to enhance histamine toxicity when present in spoiled fish by inhibiting the intestinal histamine metabolizing enzyme, including diamine oxidase [1,2].

Milkfish (*Chanos chanos*) is widely distributed throughout the Indo-Pacific region and is the second most important inland aquaculture fish in Taiwan [7,8]. This fish has been cultivated in Taiwan for more than 350 years. Taiwan’s total milkfish production is approximately 50,000–60,000 tons each year [8]. Chiou et al. [9] demonstrated that histidine at 441 mg/100 g is the most prominent free amino acid (FAA) in the white muscles of milkfish and accounts for 80% of the total FAAs in the fish. Therefore, milkfish products have become most often associated with HFP in Taiwan, including dried milkfish [6], milkfish sticks [10], and milkfish surimi [11]. In addition, our research team determined that 78% of commercial dry-salting and dried milkfish products have histamine contents greater than the 5 mg/100 g recommended value of the United States Food and Drug Administration’s (USFDA) guidelines, while 43.7% of the fish samples were found to exceed 50 mg/100 g of histamine [12].

In general, there are two major salting methods for milkfish preservation, namely, dry-salting and brine-salting. In Taiwan, the traditional processes of dry-salting and dried milkfish include scaling, back-cutting, degutting, and dry-salting with 3–12% NaCl (*w*/*w*) followed by sun-drying for 5–7 days [12]. However, the consumption of high salt levels from seafood can result in several chronic diseases, such as hypertension and cardiovascular diseases [13]. Brine-salting for fish processing may be a better method to reduce salt uptake and water loss and, thus, to reach a higher weight yield and better quality in salted fish compared to dry-salting [14]. Therefore, in recent years, brine- and light-salting milkfish has gained popularity with Taiwanese people. However, the quality of brined and dried fish is influenced by the brine concentrations and dry methods used for drying the fish [15].

There is no information of the occurrence of hygienic quality and histamine in brined and dried milkfish products, and the formation of histamine and the quality of brined and dried milkfish produced with different brine concentrations. Therefore, the main aim of this study was to monitor the bacteriological and chemical quality, including histamine content, in 20 brined and dried milkfish samples sold in retail stores in southern Taiwan. This work also aimed to examine the effects of different brine concentrations (0%, 3%, 6%, 9%, and 15%) on the bacteriological and chemical quality and histamine contents in brined and dried milkfish products during sun-drying for five days.

## 2. Materials and Methods

### 2.1. Materials

Twenty brined and dried milkfish products were collected from four retail stores in southern Taiwan, including store A (six samples), store B (five samples), store C (five samples), and store D (four samples). All brined and dried milkfish products were home-made by the farmer or manufacturer and delivered to the store for sale. Trackback information indicated that the samples collected from store A and D were processed using higher brine concentration (>10%) and longer sun-drying days (5–7 days); on the other hand, the samples of store B and C were processed using lower brine concentration (<6%) and shorter sun-drying days (3–5 days). In general, the processing of brined and dried milkfish include scaling, back-cutting, degutting, and brine-salting with 3–15% NaCl concentrations at room temperature for 1-2 h, followed by sun-drying for 3–7 days. After the samples were purchased, they were wrapped in aseptic bags, placed in an ice box, and instantly delivered to the laboratory for analysis within 6 h. The dorsal part of the commercial dried milkfish samples were cut and taken for microbiological and chemical determinations.

Sixty fresh milkfish (weights of 546 ± 11.6 g, lengths of 31.9 ± 1.2 cm) were purchased from the fish market of the city of Kaohsiung in Taiwan and transported to our laboratory within half an hour in an ice box. Once the fish samples arrived at the laboratory, they were manually scaled, back-cut, gutted, washed with clean water, and then drained.

### 2.2. Reagents

Histamine dihydrochloride, trichloroacetic acid, 2-thiobarbituric acid, and butylated hydroxytoluene were purchased from Sigma-Aldrich (St. Louis, MO, USA). Acetonitrile (LC grade) and dansyl chloride (GR grade) were purchased from E. Merck (Darmstadt, Germany).

### 2.3. Brine-Salting and Drying of Milkfish

The back-cut milkfish were brine-salted with concentrations of 3%, 6%, 9%, or 15% NaCl with a fish-to-brine ratio of 1:2 for 60 min at 20 °C, and unsalted milkfish were used as controls. After brine-salting, all milkfish samples were placed under sun light at 30–33 °C for seven hours each day for five days. Sampling analyses were conducted at days 1, 3, and 5 for sun-drying. The experiments were conducted in triplicate for each brine concentration and sampling time. The dorsal part of the fish samples was used for analysis.

### 2.4. Determination of pH Value, Moisture Content, Water Activity, and Salt Content

Ten grams of the samples was weighted and homogenized with a mixer (FastPrep-24, MP Biomedicals, Solon, OH, USA) for 2 min with 20 mL of deionized water to make a thick slurry. The pH of this slurry was determined using a digital pH meter (Mettler Toledo FE20/EL20, Schwerzenbach, Switzerland). The moisture of each sample (1–3 g) was measured using the oven-dry method at 105.0 ± 1.0 °C for drying, followed by the determination of the sample weight until a constant weight was achieved. Water activity was determined by an Aqualab 4TE (Decagon Devices, Pullman, WA, USA) at 25 °C. The salt (NaCl) content was determined using Mohr’s titration method [16].

### 2.5. Determination of Total Volatile Basic Nitrogen (TVBN) and Thiobarbituric Acid (TBA)

The TVBN values were measured using Conway’s dish method as described by Cobb et al. [17]. Five grams of the minced samples was homogenized with 45 mL of 6% trichloroacetic acid (TCA; Sigma-Aldrich, St. Louis, MO, USA). After the extract was filtered, saturated K_2_CO_3_ was added to the filters. The released TVBN was absorbed by boric acid and then titrated with 0.02 N HCl, while the TVBN value was expressed in milligrams per 100 g fish sample. The TBA values were determined by the modified method of Faustman et al. [18]. Briefly, 20 g of dried milkfish sample was added into a tube containing 180 mL of deionized water and then homogenized with a mixer for 3 min. Twelve milliliters of 0.1 M TBA reagent in 0.2% HCl and 0.15 mL of 0.2% butylated hydroxytoluene (BHT) in 95% ethanol were added into 2 mL of the homogenate and then mixed well. The mixtures were heated in a water bath at 90 °C for 20 min and then filtered, and the absorbance of the filtrates was detected using a spectrophotometer (UV-1201, Shimazu, Tokyo, Japan) at 532 nm. The TBA values in the fish samples are expressed in milligrams of malondialdehyde (MDA) per kilogram.

### 2.6. Microbiological Analysis

Twenty-five grams of the minced samples was homogenized with 225 mL of sterile 0.85% (*w*/*v*) physiological saline in a sterile blender at a 1200 rpm speed for 2 min. The homogenate was serially diluted with a sterile physiological saline for 1:10 (*v*/*v*) dilutions. With regard to spread plate counting, 0.1 mL of the dilutes was spread on aerobic plate count (APC) agar (Difco, BD, Sparks, MD, USA) with 0.5% NaCl and then incubated at 30 °C for 24–48 h. After the bacterial colonies grown on the plate were counted, the data were expressed as log_10_ colony forming units (CFUs) per gram. The levels of coliform and *Escherichia coli* in the milkfish samples were performed according to the three-tube most probable number (MPN) method as described by the FDA [19].

### 2.7. Histamine Analysis

Histamine dihydrochloride (82.8 mg) was dissolved in 50 mL of 0.1 M HCl and used as the working solution, and the final concentration of histamine (free base) was 1.0 mg/mL. Five grams of the ground milkfish samples were homogenized with 20 mL of 6% cold trichloroacetic acid (TCA) using a Polytron PT-MR 3100 homogenizer for 3 min. The homogenates were collected via centrifugation at 4500× *g* for 8 min at 7 °C and filtered through Advantec Toyo No. 2 filter paper. The filtrates were diluted up to 50 mL with a 6% TCA solution. For the derivatization reaction of histamine, 1 mL aliquots of the TCA extract of each sample and histamine standard solution were derivatized with dansyl chloride using the method of Chen et al. [3] with some modifications. Briefly, 0.2 mL of 2 M sodium hydroxide and 0.3 mL of saturated sodium bicarbonate were added to 1 mL aliquots of the TCA extract of each sample and the histamine standard solution. The solution was added to 2 mL of 1% dansyl chloride solution dissolved in acetone, mixed by a vortex mixer, and left to stand at 40 °C for 45 min. After the reaction, 100 µL of ammonia was added to terminate the derivatization reaction. Acetonitrile was added to a final volume of 5 mL and the solution was centrifuged (10,000× *g*, 5 min, 4 °C). After the supernatants were filtered through 0.22 μm membrane filters, 20 μL of the filtrates were injected into high-performance liquid chromatography (HPLC). The histamine levels in each milkfish sample were analyzed by HPLC (Hitachi, Tokyo, Japan) equipped with a LiChrospher 100 RP-18 reversed-phase column (5 μm, 125 × 4.6 mm, E. Merck, Damstadt, Germany) and a UV-Vis detector (Model L-4000, Hitachi, wavelength at 254 nm). The mobile phase consisted of eluent A (acetonitrile) and eluent B (water). At the beginning, eluents A and B at a ratio of 50:50 (*v*/*v*) were applied for 19 min, followed by a linear gradient with an increase of eluent A up to 90% during the next minute. In the final 10 min, the eluent A and B mix was set to a linear decrease to 50:50 (*v*/*v*). The flow rate was 1.0 mL/min. Validation of the histamine analysis method including inter- and intra-day repeatability (expressed as % and relative standard deviation, RSD) was determined by fortifying homogenized dried milkfish meats with 1.0, 5.0, and 10 mg/100 g of standard histamine. Each spiked amount was extracted and derivatized with dansyl chloride using the above procedure in triplicate, including a blank test to evaluate the average recovery.

### 2.8. Statistical Analysis

One-way analysis of variance (ANOVA) and Tukey’s pairwise comparison tests were performed within the 95% confidence interval. Pearson correlation was carried out to determine relationships between pH, moisture, water activity, salt content, TVBN, APC, coliform, and histamine contents in the brined and dried milkfish samples. All statistical analyses were carried out using the Statistical Package for Social Sciences (SPSS) Version 16.0 for Windows (SPSS Inc., Chicago, Il, USA), and *p* < 0.05 was used to consider significant deviation.

## 3. Results and Discussion

### 3.1. Chemical and Bacteriological Quality of the Brined and Dried Milkfish Samples

For all 20 brined and dried milkfish samples collected from the four retail stores, the pH, moisture, water activity, salt content, TVBN, APC, coliform, and histamine ranged from 5.67 to 6.05, 38.27% to 69.78%, 0.89 to 0.98, 0.16% to 4.37%, 8.86 to 19.88 mg/100 g, 3.51 to 8.25 log CFU/g, <3 to >2400 MPN/g, and 0.16 to 301 mg/100 g, respectively (Table 1). *E. coli* was not detected in any milkfish samples. Store A samples had significantly lower (*p* < 0.05) mean water activity (0.94) than did samples collected from the other three stores, while the mean salt content (3.23%) in store A samples was higher (*p* < 0.05) than the others (Table 1). Moreover, the mean TVBN and APC values in store B samples (16.06 mg/100 g and 6.62 log CFU/g, respectively) and store D samples (16.82 mg/100 g and 6.33 log CFU/g, respectively) were markedly higher (*p* < 0.05) than those samples obtained from the other two stores, while the mean coliform level (356 MPN/g) in store D samples were higher than that of the other stores (Table 1). The highest mean histamine content of 79 mg/100 g was obtained from five samples from store B, followed by store D with a mean of 4.9 mg/100 g of histamine.

In this study, the proportion of the 20 brined and dried milkfish samples that did not meet the 6.47 log CFU/g Taiwanese regulatory standard for APC was 35% (7/20). Therefore, brined and dried milkfish manufacturers may need to be more careful with hygienic handling or processing in their preparation of brined and dried milkfish products. The distribution of histamine contents in the brined and dried samples is shown in Table 2. Five samples (25%, 5/20) failed to meet the 5 mg/100 g level of histamine, the allowable limit by the USFDA for scombroid fish and/or products, while two (10%) had 69 and 301 mg/100 g of histamine, greater than the potential toxicity level (50 mg/100 g). According to information by Bartholomev et al. [20], which showed that fish with histamine levels >100 mg/100 g could result in illness and health hazards if ingested by humans, one sample with 301 mg/100 g of histamine could have caused disease symptoms if consumed (Table 2). In contrast, our previous research showed that 78.1% (25 samples) and 43.7% (14 samples) of 32 dry-salted and dried milkfish products contained more than 5 mg/100 g and 50 mg/100 g of histamine, respectively [21].

High levels of histamine have been found in various types of milkfish implicated in HFP. Our research group detected 61.6 mg/100 g of histamine in dried milkfish products that were implicated in an incident of HFP [6]. Two fried milkfish sticks implicated in a poisoning incident contained 86.6 mg/100 g and 235.0 mg/100 g of histamine [10]. The high content of histamine (i.e., 91.0 mg/100 g) in a suspected milkfish surimi product could be the etiological factor for this fish-borne poisoning in Taiwan [11]. Therefore, it is also very important for people, especially those from the Indo-Pacific region, such as the Philippines, Indonesia, and Taiwan, to be aware that milkfish products could become a hazardous food item, causing histamine poisoning.

Pearson correlation was conducted to determine if there existed any relationship among the pH, moisture, water activity (a_w_), salt content, TVBN, APC, coliform, and histamine contents of the tested 20 samples. In general, positive correlations existed between moisture and a_w_ (*r*, correlation coefficient = 0.81, *p* < 0.05), TVBN and APC (*r* = 0.76, *p* < 0.05), APC and histamine (*r* = 0.71, *p* < 0.05), and histamine and TVBN (*r* = 0.76, *p* < 0.05). However, negative correlations were noted between moisture and salt content (*r* = −0.73, *p* < 0.05), and a_w_ and salt content (*r* = −0.76, *p* < 0.05).

### 3.2. Effect of Brine Concentrations on the Quality of Brined and Dried Milkfish

**Figure 1:**
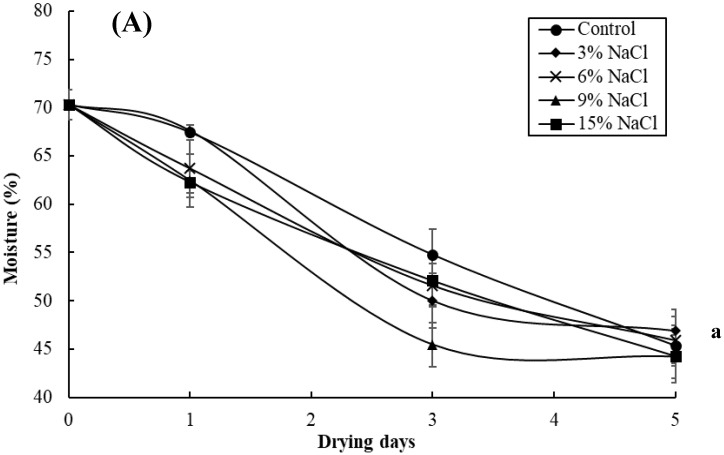

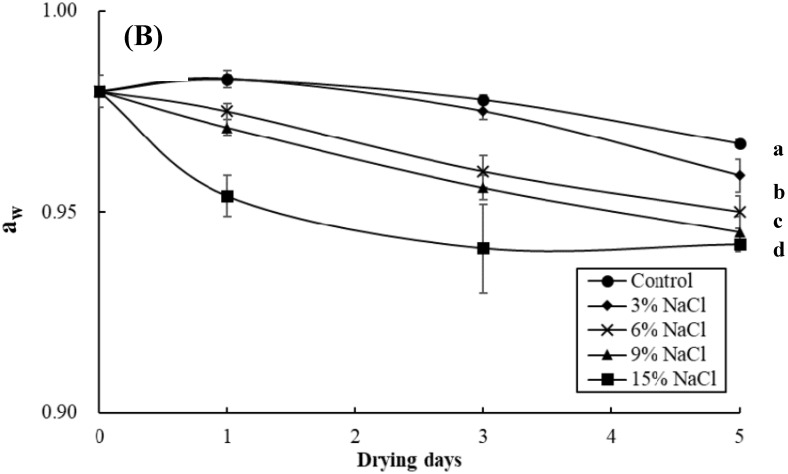
Changes in the moisture (**A**) and water activity (a_w_) (**B**) in the milkfish samples as a result of brine-salting with 0% (control), 3%, 6%, 9%, and 15% NaCl during sun-drying. Each value represents the mean ± SD of three replications. Different lower letters indicate significant differences (*p* < 0.05) within the data at the end of the sun-drying period.

Changes in the moisture and water activity (a_w_) of the milkfish samples pre-immersed in different brine concentrations (i.e., 0%, 3%, 6%, 9%, and 15%) during a sun-drying period of five days are shown in Figure 1. The initial moisture of the fish samples was 70.3%, while the moisture of all fish samples rapidly decreased with increasing drying time. At the end of the drying period, the moisture content in all of the samples ranged from 44.2% to 46.9%, and no significant differences (*p* > 0.05) were observed among the samples of the various brine concentrations and control samples (Figure 1A). For all fish samples with an initial a_w_ value of 0.985, the a_w_ values gradually decreased with an increase in the drying time and reduced to 0.967 in the control sample, 0.959 in the 3% NaCl sample, 0.950 in the 6% NaCl sample, 0.945 in the 9% NaCl sample, and 0.942 in the 15% NaCl sample at the end of the sun-drying period (Figure 1B). It was found that the milkfish samples with higher brine concentrations had lower a_w_ values (*p* < 0.05).

**Figure 2:**
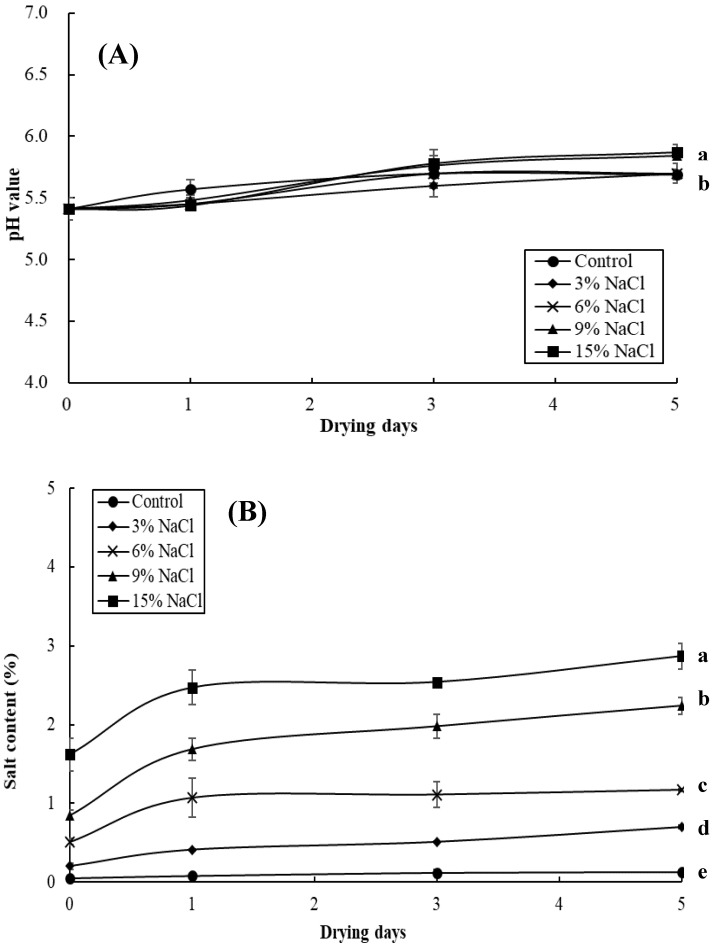
Changes in the pH (**A**) and salt content (**B**) of the milkfish samples as a result of brine-salting with 0% (control), 3%, 6%, 9%, and 15% NaCl during sun-drying. Each value represents the mean ± SD of three replications. Different lower letters indicate significant differences (*p* < 0.05) within the data at the end of the sun-drying period.

Changes in the pH and salt content of the milkfish samples pre-immersed in different brine concentrations (i.e., 0%, 3%, 6%, 9%, and 15%) over a sun-drying period of five days are presented in Figure 2. The pH values of the milkfish samples slightly increased from the initial reading of 5.41 to 5.69 for the control sample, 5.70 for the 3% and 6% NaCl samples, 5.87 for the 9% NaCl sample, and 5.89 for the 15% NaCl sample at the end of the sun-drying period. The increase in the pH for all of the group samples may be due to the formation of basic components, including ammonia, trimethylamine, and other amines by bacterial spoilage [22]. Moreover, the final pH values of the 9% and 15% NaCl samples were higher (*p* < 0.05) than those of the control and the 3% and 6% NaCl samples (Figure 2A). As shown in Figure 2B, the salt content in the fish sample slightly increased from 0.05% to 0.13% in the control sample, 0.20% to 0.70% in the 3% NaCl sample, 0.51% to 1.17% in the 6% NaCl sample, 0.85% to 2.24% in the 9% NaCl sample, and 1.62% to 2.87% in the 15% NaCl sample after give days of sun-drying. The results also show that the milkfish samples pre-immersed in a higher brine concentration had a higher salt content (*p* < 0.05).

**Figure 3:**
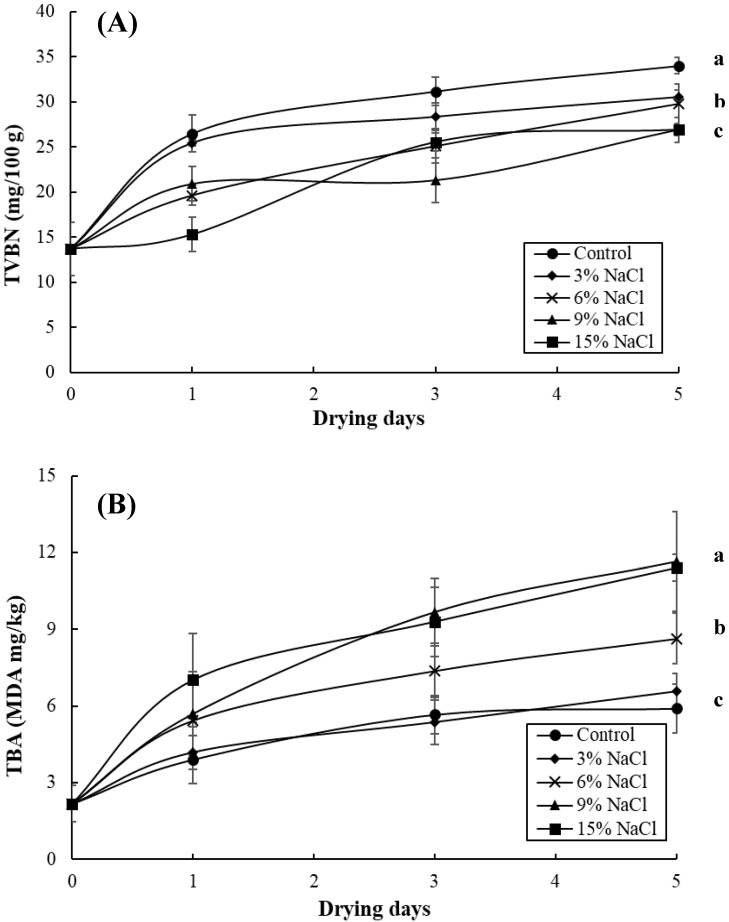
Changes in total volatile basic nitrogen (TVBN) (**A**) and thiobarbituric acid (TBA) (**B**) in the milkfish samples as a result of brine-salting with 0% (control), 3%, 6%, 9%, and 15% NaCl during sun-drying. Each value represents the mean ± SD of three replications. Different lower letters indicate significant differences (*p* < 0.05) within the data at the end of the sun-drying period.

Figure 3 shows the changes in the TVBN and TBA values in the milkfish samples pre-immersed in different brine concentrations (i.e., 0%, 3%, 6%, 9%, and 15%) during a sun-drying period of five days. Initially, the milkfish samples had 13.7 mg/100 g of TVBN, and subsequently, the TVBN content in all fish samples increased gradually while drying, reaching 34.0 mg/100 g for the control sample, 30.5 mg/100 g for the 3% NaCl sample, 29.76 mg/100 g for the 6% NaCl sample, 27.0 mg/100 g for the 9% NaCl sample, and 26.9 mg/100 g for the 15% NaCl sample at the end of the sun-drying period. Thus, the highest TVBN level was detected in the control sample, followed by the 3% and 6% NaCl samples, and the lowest levels were observed for the 9% and 15% NaCl samples (*p* < 0.05) (Figure 3A). Connell [23] revealed that the increase in TVBN is due to the production of volatile basic compounds, including ammonia, trimethylamine and dimethylamine, via decomposition by autolytic enzymes and spoilage bacteria. Moreover, Nooralabettu [15] demonstrated that the addition of NaCl addition in Bombay duck can decrease autolytic enzyme activity in fish meat. An increase in salt content above 1% in fish can have an inhibitory effect on the bacteria associated with fish spoilage [24]. Consequently, the high content of TVBN in the unsalted samples (i.e., the control sample) obtained in this study was probably due to the increasing decomposition by enzymes and spoilage bacteria with the lack of salt’s inhibitory effect.

Thiobarbituric acid (TBA), a measure of MDA as a secondary lipid oxidation product, is one of the most widely used indicators for the assessment of food lipid oxidation [25]. Initially, the TBA values for the control and brined samples were 2.18 MDA mg/kg. The value of TBA in all of the samples increased during the sun-drying period, reaching 5.9 MDA mg/kg for the control sample, 6.5 MDA mg/kg for the 3% NaCl sample, 8.6 MDA mg/kg for the 6% NaCl sample, 11.5 MDA mg/kg for the 9% NaCl sample, and 11.4 MDA mg/kg for the 15% NaCl sample at the end of the sun-drying period. In contrast to TVBN, the highest levels of TBA were observed in the 9% and 15% NaCl samples, followed by the 6% NaCl sample, and the lowest TBA level was detected in the control and 3% NaCl samples (*p* < 0.05) (Figure 3B). Yanar et al. [26] also reported that hot-smoked tilapia samples treated with a 15% brine concentration contained very high levels of TBA. Sodium chloride can promote lipid oxidation, while sodium ions may replace iron from myoglobin, thereby resulting in free iron ions for the catalysis of lipid oxidation [26,27]. Therefore, the results in this study reveal that the high TBA values in the samples prepared with 9% and 15% brine concentrations may be attributed to the addition of NaCl by accelerating the rate of lipid oxidation. In addition, when seafood is dried by exposure to sunlight, lipids can be oxidized and low molecular weight carbonyl components can be produced [28]. The results of this study are in agreement with a previous study reporting that the TBA values of dried yellow corvina increased rapidly during sun-drying [28].

**Figure 4:**
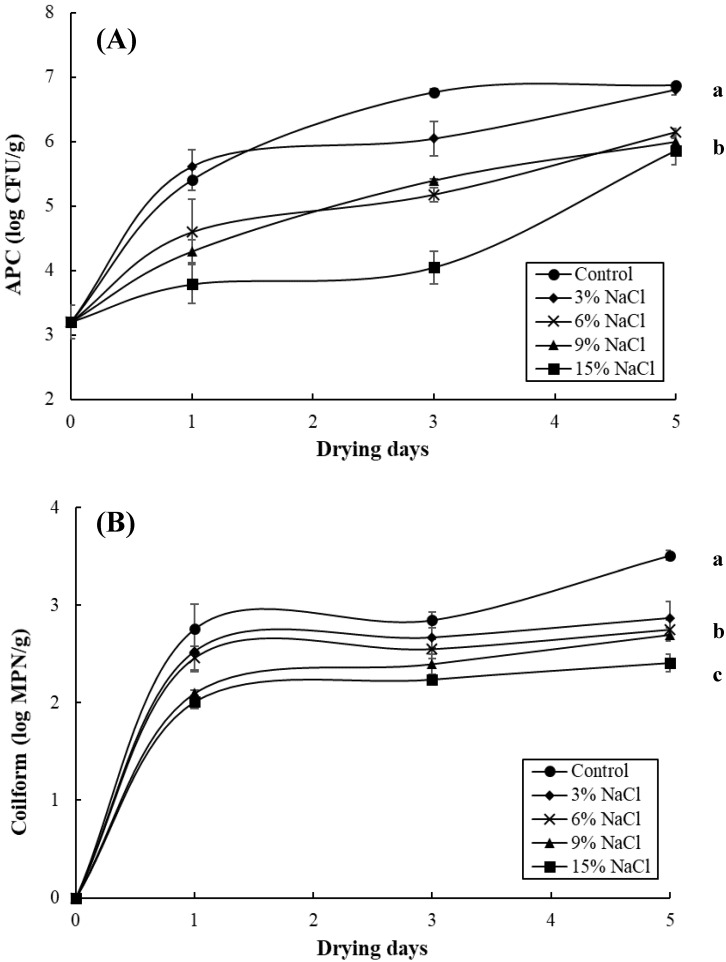
Changes in aerobic plate count (APC) (**A**) and coliform (**B**) in the milkfish samples as a result of brine-salting with 0% (control), 3%, 6%, 9%, and 15% NaCl during sun-drying. Each value represents the mean ± SD of three replications. Different lower letters indicate significant differences (*p* < 0.05) within the data at the end of the sun-drying period.

Figure 4 shows the changes in APC and coliform bacteria in the milkfish samples pre-immersed in different brine concentrations (i.e., 0%, 3%, 6%, 9%, and 15%) over a sun-drying period of five days. The APC numbers of the milkfish sample gradually increased from the initial population of 3.21 to 6.88 log CFU/g for the control sample, 6.81 log CFU/g for the 3% NaCl sample, 6.15 log CFU/g for the 6% NaCl sample, 6.0 log CFU/g for the 9% NaCl sample, and 5.86 log CFU/g for the 15% NaCl sample at the end of the sun-drying period. Thus, the APC bacteria detected in the control and 3% NaCl samples were markedly higher (*p* < 0.05) than those of other brine concentration samples (Figure 4A) and exceeded the 6.47 log CFU/g Taiwanese regulatory standard. Similar to the APC population, the growth of coliform in this fish samples was considerably faster in the unsalted (control) sample than in the other brined samples (*p* < 0.05). The coliform counts in the control, 3%, 6%, 9%, and 15% NaCl samples increased to 3.51, 2.87, 2.75, 2.70, and 2.41 log MPN/g, respectively, at the end of the sun-drying period (Figure 4B). These results are in agreement with our previous report, in which the APC and coliform levels of dry-salted and sun-dried milkfish samples decreased with increasing salt concentrations [21]. A similar finding was also reported by Yang et al. [14], who found that higher brine-salting could inhibit the growth of bacteria in grass carp. Moreover, higher brine concentrations (>6%) in the milkfish samples obviously had a repressive action on microbiological growth in this study, indicating that salt content is able to inactivate or inhibit bacteria.

**Figure 5:**
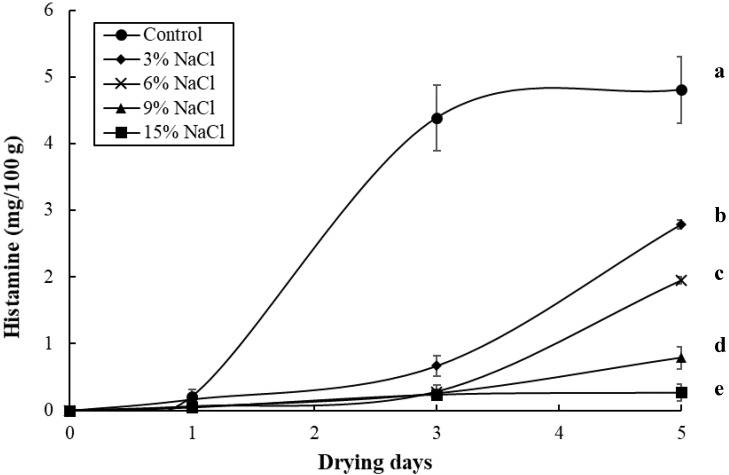
Changes in the histamine of milkfish samples as a result of brine-salting with 0% (control), 3%, 6%, 9%, and 15% NaCl during sun-drying. Each value represents the mean ± SD of three replications. Different lower letters indicate significant differences (*p* < 0.05) within the data at the end of the sun-drying period.

Figure 5 shows that the histamine content in the control sample increased gradually during the sun-drying period, reaching 4.8 mg/100 g by the end. On the other hand, the histamine contents in the 3%, 6%, 9%, and 15% NaCl samples only slightly increased during the sun-drying period, reaching 2.8, 2.0, 0.79, and 0.27 mg/100 g, respectively, by the end. In conclusion, the histamine content observed in the control sample was markedly higher (*p* < 0.05) than that of the other brine concentrations samples (Figure 5). These results agree with the previous research of Hwang et al. [21], where high contents of histamine at 67 mg/100 g were found in unsalted dried milkfish samples via sun-drying. The low levels of histamine (<2.8 mg/100 g) detected in the salted samples (>3% NaCl) in this study may be due to the growth reduction of histamine-forming bacteria by the preservative effect of salt, indicating that the addition of salt could be effective in reducing or inhibiting histamine accumulation. In our previous study, high levels of a_w_, moisture, TVBN, APC, and histamine were detected in unsalted dried milkfish samples produced by sun-drying; therefore, dried milkfish producers should be aware that dried milkfish with low salt and sun-drying periods could become a vehicle for histamine poisoning [21]. Similarly, since high levels of TVBN (>30 mg/100g), APC (>6.81 log CFU/g), and histamine (>2.8 mg/100 g) were observed in the unsalted and 3% NaCl samples during the sun-drying period, brined and dried milkfish manufacturers should pay attention to the fact that dried milkfish brined with a low amount of salt (<3% NaCl) and a sun-drying period could lead to worse hygienic quality and potential hazards, such as food poisoning. However, the samples with higher brine concentrations (>9% NaCl) had higher TBA values (>11.4 MDA mg/kg) (Figure 3B). With regard to an assessment of APC, TBA, TVBN, and histamine, this study suggests that dried milkfish brined with a 6% NaCl addition has better chemical and bacteriological quality.

Pearson correlation was conducted to determine if there existed any relationship among the moisture, a_w_, pH, salt content, TVBN, TBA, APC, coliform, and histamine contents of the samples at the end of the sun-drying period. In general, positive correlations existed between APC and a_w_ (*r* = 0.95, *p* < 0.05), APC and histamine (*r* = 0.88, *p* < 0.05), coliform and a_w_ (*r* = 0.93, *p* < 0.05), coliform and histamine (*r* = 0.90, *p* < 0.05), a_w_ and TVBN (*r* = 0.88, *p* < 0.05), salt content and TBA (*r* = 0.85, *p* < 0.05), a_w_ and histamine (*r* = 0.89, *p* < 0.05), and histamine and TVBN (*r*=0.86, *p* < 0.05). However, negative correlations were noted between salt content and APC (*r* = −0.89, *p* < 0.05), salt content and coliform (*r* = −0.90, *p* < 0.05), salt content and a_w_ (*r* = −0.92, *p* < 0.05), a_w_ and TBA (*r* = −0.90, *p* < 0.05), and salt content and histamine (*r* = −0.95, *p* < 0.05).

## 4. Conclusions

This study, aimed at investigating the hygienic quality of 20 brined and dried milkfish products, revealed that the APC numbers in seven samples (35%) exceeded the 6.47 log CFU/g Taiwanese regulatory standard. Moreover, 25% of the tested samples had histamine contents greater than the 5 mg/100 g recommended by the USFDA in their guideline levels, and 10% (2/20) of the fish samples had >50 mg/100 g of histamine. After the consumption of these samples, histamine fish poisoning could occur. In addition, the chemical and bacteriological quality of the brined and dried milkfish pre-immersed in various brine concentrations during a sun-drying period were observed in this study. Although the samples prepared with higher brine concentrations presented a retarded APC growth rate and a reduced formation of TVBN and histamine, as compared with the control sample, they produced higher TBA values. It is suggested that 6% NaCl for brined milkfish is the optimal condition for maintaining the quality of brined and dried milkfish. Our results could suggest that application of brine concentration information is effective in controlling quality and enhancing the safety of brined and dried milkfish products.

## Figures and Tables

**Table 1:** pH, moisture, water activity, salt content, total volatile basic nitrogen (TVBN), aerobic plate count (APC), coliform, *Escherichia coli,* and histamine values in brined and dried milkfish products.

Sample Sources	Number of Samples	pH	Moisture (%)	Water Activity	Salt Content (%)	TVBN (mg/100 g)	APC (log CFU/g)	Coliform (MPN/g)	*E. coli* (MPN/g)	Histamine (mg/100 g)
A	6	5.74~5.83 (5.78 ± 0.04) ^A^	38.27~65.37 (51.78 ± 9.28) ^B^	0.89~0.97 (0.94 ± 0.03) ^B^	2.47~4.37 (3.23 ± 0.78) ^A^	8.86~17.36 (12.79 ± 3.25) ^B^	3.51~7.65 (5.50 ± 1.05) ^B^	<3~70 (42 ± 25) ^B^	<3	0.34~4.9 (1.3 ± 1.8) ^C^
B	5	5.67~6.05 (5.76 ± 0.17) ^A^	52.31~60.18 (55.41 ± 3.23) ^AB^	0.97~0.98 (0.98 ± 0.01) ^A^	0.16~1.88 (0.80 ± 0.77) ^C^	13.86~19.32 (16.06 ± 2.08) ^A^	5.87~8.03 (6.62 ± 0.83) ^A^	<3~40 (25 ± 13) ^B^	<3	0.62~301 (79 ± 57) ^A^
C	5	5.80~5.95 (5.87 ± 0.07) ^A^	54.68~69.78 (64.17 ± 5.95) ^A^	0.97~0.98 (0.98 ± 0.01) ^A^	0.61~1.02 (0.86 ± 0.18) ^C^	10.85~13.93 (12.35 ± 1.39) ^B^	4.18~5.92 (5.32 ± 0.66) ^B^	<3~240 (90 ± 130) ^B^	<3	0.16~0.31 (0.20 ± 0.11) ^C^
D	4	5.75~5.82 (5.78 ± 0.03) ^A^	51.07~54.12 (52.46 ± 1.26) ^B^	0.95~0.96 (0.96 ± 0.01) ^AB^	1.47~1.80 (1.68 ± 0.15) ^B^	15.40~19.88 (16.82 ± 2.66) ^A^	5.80~8.25 (6.33 ± 0.60) ^A^	20~>2400 (356 ± 47) ^A^	<3	0.24~19 (4.9 ± 4.8) ^B^

The values in parentheses represent the mean ± standard deviation (SD). Values in the same column with different letters are statistically different (*p* < 0.05). CFU, colony forming unit; MPN, most probable number.

**Table 2:** Distribution of the histamine content in the 20 brined and dried milkfish products.

Content of Histamine (mg/100 g)	Brined and Dried Milkfish Products	
	Number of Samples	% of Samples
<4.9	15	75
5.0–49.9	3	15
50.0–99.9	1	5
>100	1	5
Total	20	100
